# Influence of Graphene Nanoplatelets and Post-Curing Conditions on the Mechanical and Viscoelastic Properties of Stereolithography 3D-Printed Nanocomposites

**DOI:** 10.3390/polym16192721

**Published:** 2024-09-26

**Authors:** Khalid Haj Ahmad, Zurina Mohamad, Zahid Iqbal Khan

**Affiliations:** 1 Enhanced Polymer Research Group, Faculty of Chemical and Energy Engineering, Universiti Teknologi Malaysia, Johor Bahru, Skudai 81310, Malaysia; 2 College of Engineering, Alfaisal University, P.O. Box 50927, Riyadh 11533, Saudi Arabia

**Keywords:** photopolymer, nanocomposites, 3D printing, nanoindentation, stereolithography, post-cured

## Abstract

This study presents an innovative approach to improving the mechanical and viscoelastic properties of 3D-printed stereolithography (SLA) nanocomposites by incorporating graphene nanoplatelets (xGNP) into photopolymer matrices. Utilizing an SLA 3D printer, photopolymer formulations with xGNP concentrations of up to 0.25 wt% were successfully produced. Post-print curing was carried out using two different methods: ultraviolet (UV) curing and high-temperature curing at 160 °C. Mechanical characterization using nanoindentation showed a significant increase in elastic modulus by 104% and an increase in hardness by 85% for nanocomposites containing 0.25 wt% xGNP. Furthermore, dynamic mechanical analysis (DMA) revealed a 39% improvement in storage modulus for samples without post-curing and an improvement of approximately 30% for samples subjected to high-temperature curing. These significant improvements highlight xGNP’s potential to not only increase the performance of SLA 3D-printed components but also streamline the manufacturing process by reducing or eliminating energy-intensive post-curing steps. This innovative integration of graphene nanoplatelets paves the way for the production of high-performance, functional 3D-printed products and offers significant advances for various industries with a high impact. The results highlight the transformative role of nanomaterials in additive manufacturing and position this work at the forefront of materials science and 3D printing technology.

## 1. Introduction

Three-dimensional printing, also known as 3D printing, is a versatile and rapidly growing field. It has a variety of applications in prototyping, jewelry, dentistry, pharmaceuticals, and custom manufacturing [1]. It uses the layer-by-layer construction of objects of arbitrary geometry from a computer-aided design source file. Its flexibility and reproducibility make 3D printing one of the most promising techniques for rapid prototyping and customized manufacturing. Stereolithography is one of the important 3D printing processes [2]. It produces parts by polymerizing reactive resin using photoinitiators activated by the light source, a laser beam that moves to cure the printed layer. This method can be used to produce high-resolution parts with a good surface quality [3].

Photocurable resins containing acrylate, methacrylate, or epoxy groups represent a crucial class of thermosets known for their rapid curing under radiation. Their beneficial properties include chemical and corrosion resistance, high modulus, and thermal and dimensional stability. Consequently, these resins find extensive application in industries such as aerospace, biomedicine, and microelectronics, particularly in stereolithography processes [4]. Nanofillers can provide a wide range of materials with enhanced properties and multifunctionality for 3D printing to a wide range of materials, such as electrical and thermal conductivities and mechanical strength [5]. Polymer nanocomposites show significant property improvements at much lower loadings than polymer composites with conventional micron-scale fillers (such as glass or carbon fibers), which ultimately leads to lower component weight and can simplify processing. Furthermore, the improvements in multifunctional properties enabled by nanocomposites could create new applications for polymers [6]. 

While the primary aim of incorporating nanofillers is typically to enhance mechanical properties, certain studies have shown unintentional degradation instead [7,8]. For example, the introduction of graphene oxide into a commercially available acrylate-based photopolymer resulted in reduced elastic modulus and tensile strength in 3D-printed samples [9,10]. Similarly, the addition of hexagonal boron nitride nanoparticles to another commercially available acrylate-based photopolymer resulted in reduced microhardness and compressive strength in 3D-printed samples [11]. Nonetheless, other studies have shown that improvements in the mechanical properties of 3D-printed nanocomposites can be achieved, albeit at low graphene concentrations, typically up to 0.1 wt% [12,13]. Furthermore, the glass transition temperature (T_g_) is a critical thermal property that is influenced by the cross-linking density of the polymer network. In general, higher cross-link density correlates with increased T_g_, as it limits the mobility of the polymer chains and increases the stiffness and thermal stability of the material. Recent studies, such as those examining polyimide-crosslinked silica aerogels, confirm this connection and highlight that increased cross-linking density increases T_g_ by restricting chain movement and reducing free volume within the polymer matrix [14]. Similarly, research on poly(acrylic acid) further supports the correlation between cross-link density and T_g_ increase due to reduced free volume and increased chain stiffness [15]. However, the introduction of nanofillers such as graphene nanosheets (xGNP) can complicate this relationship and potentially alter the T_g_ due to interactions that can induce plasticizing effects or create heterogeneous cross-linking networks. These findings highlight the existing limitations and inconsistencies in the application of nanofillers, particularly graphene, within photopolymers signal an urgent need for further research to develop formulations that not only overcome these challenges but also maximize the mechanical and viscoelastic benefits at higher concentrations.

The aim of this study is to investigate the novel integration of graphene nanoplatelets into photopolymers, focusing on their transformative influence on the mechanical and viscoelastic properties of 3D-printed nanocomposites. By studying how these nanoplatelets impact the printability of the material, we uncover the potential to significantly improve the mechanical strength of printed components. Such advances could lead to a breakthrough reduction or even elimination of the need for extensive post-curing processes, resulting in significant time and energy savings. This innovative approach not only improves material performance but also pushes the boundaries of 3D printing technology. While post-curing processes such as UV and thermal curing are known to significantly improve the cross-linking density and thus the mechanical properties of 3D-printed photopolymers, the addition of graphene nanoplatelets offers significant advantages. The incorporation of xGNP not only strengthens the polymer matrix through efficient load transfer and improved energy dissipation but also improves the initial mechanical and viscoelastic properties even before post-curing. This fundamental improvement lays the foundation for further post-cure optimization and highlights the complementary relationship between xGNP addition and post-cure treatments to achieve superior material performance.

## 2. Materials and Methods

### 2.1. Materials

The commercially available acrylate-based photopolymer resin Formlabs Clear (FLGPCL04) was purchased from Formlabs (Somerville, MA, USA). Graphene nanoplatelets (xGNP), C500, with a surface area of 500 m^2^/g and a particle size of <2 μm, were purchased from XG Sciences, Lansing, MI, USA.

### 2.2. Sample Preparation

Photopolymer formulations were prepared by adding xGNP (C-500) to the photopolymer resin under shear mixing for 5 min, followed by ultrasonication for 20 min. The concentrations of xGNP added during the preparation of the nanocomposites were 0.05 wt%, 0.10 wt%, 0.15 wt%, and 0.25 wt%. These different concentrations were chosen to systematically study the effect of graphene content on the mechanical and viscoelastic properties of the 3D-printed nanocomposites, thereby identifying the optimal concentration to improve the performance of the material. During the printing experiments, it was observed that increasing the concentration of graphene nanoplatelets beyond 0.25 wt% resulted in poor interlayer adhesion and thus printing defects. This was due to the agglomeration of xGNP at higher concentrations, which disturbed the uniformity and flow of the photopolymer resin and affected the adhesion between successive layers during the SLA printing process. The photopolymer mixture was used in the SLA 3D printing process. Post-curing of 3D-printed nanocomposite samples was performed using two different methods: UV and high-temperature curing. UV curing was carried out in the FormCure UV chamber (Formlabs Inc., Somerville, MA, USA) for 15 min at room temperature. The high-temperature curing took place in an oven at 160 °C for 15 min.

### 2.3. Characterization Methods

The nanoindentation platform Nano Test 3 (Product of Micromaterials, Wrexham, UK) was used to determine the elastic modulus and hardness values of the printed samples. The load was measured as a function of the depth deformation. A Berkovich-type diamond indenter with a maximum load of 20 mN was used. The experiment was carried out in the following order: (1) approaching the surface, (2) loading to the peak load of 20 mN at a rate of 5 mN/s, (3) holding the indenter at the peak load for 30 s, and (4) discharge from the peak load at a rate of 20 mN/s. The holding step was installed to eliminate the effect of creep. Elastic modulus and hardness values were determined by analysis with Nanotest software version 30.41. Nanoindentation is widely used to determine the elastic modulus and hardness of polymer materials [16]. For both elastic modulus and hardness testing, each experimental condition was evaluated using at least three duplicate samples to ensure the accuracy and reproducibility of the results. The values reported represent the average of these measurements, and standard deviations are included to indicate variability between samples. This approach ensures that the data presented is robust, reliable, and reflects consistent material performance across different samples. Differential scanning calorimetry (DSC) tests were carried out to evaluate and verify the thermal properties of the photopolymer and its nanocomposite. The instrument used for this analysis was a Hitachi DSC 7020 instrument from Tokyo, Japan, with a heating rate of 10 °C/min under nitrogen. Samples of each formulation were tested at ambient temperatures up to 300 °C under a nitrogen atmosphere. Viscoelastic properties were measured using dynamic mechanical analysis (DMA) performed with a Perkin Elmer DMA8000 with a frequency of 1 Hz and a temperature sweep from 40 °C to 200 °C at 5 °C/min. DMA was used to evaluate the storage modulus and T_g_ of the samples, paying particular attention to how cross-linking density and nanofiller distribution affect these properties. It is important to consider that cross-linking density generally correlates with increased T_g_, the interaction between nanofillers and the polymer matrix. 

## 3. Results and Discussion

### 3.1. Three-Dimensionally Printed Photopolymer and Its Nanocomposites

The printability of photopolymer nanocomposites containing xGNP (C-500) was assessed using an SLA 3D printer. Various concentrations of C-500, starting at 0.05 wt%, were 3D-printed at a resolution of 25 μm to determine the maximum printable concentration of the nanofiller. It was found that photopolymer could be printed with a maximum of 0.25 wt% C-500. However, at higher concentrations, the printing process failed due to poor adhesion between successive layers, which is due to a lower light penetration depth [17]. At concentrations above 0.25% by weight, the printing process encountered significant challenges, primarily due to poor adhesion between successive layers. This was attributed to the excessive presence of graphene nanoplatelets (xGNP), which resulted in agglomeration and uneven distribution within the photopolymer matrix. The agglomerated nanoplatelets disrupted the uniform bonding between the printed layers and created weak points that compromised the structural integrity of the printed parts. Furthermore, higher xGNP concentrations changed the viscosity and rheological properties of the resin, affecting its flow and curing behavior and thus further affecting the interlayer adhesion. To ensure successful printing and maintain good layer bonding, the xGNP concentration was optimized to 0.25 wt% or less to balance improved mechanical properties with printability. The mechanical characterization of the nanocomposites showed significant improvements in elastic modulus and hardness with the addition of xGNP, which were further enhanced by post-curing at 160 °C. Notably, the incorporation of 0.25 wt% xGNP resulted in an increase of 104% in elastic modulus and 85% in hardness compared to the control samples, which is attributed to the effective dispersion of graphene nanosheets within the polymer matrix. This distribution enables better load transfer and energy dissipation within the material, thus ensuring a significant improvement in mechanical properties, regardless of post-curing effects. Post-curing, particularly at 160 °C, further enhances these properties by increasing the cross-linking density. However, the fundamental improvement is fundamentally due to the presence of xGNP. This highlights the synergistic relationship where the addition of xGNP provides a critical base improvement and post-curing serves to maximize these effects.

### 3.2. Viscoelastic Properties

DMA provides valuable insights into the viscoelastic behavior of materials by measuring parameters such as storage modulus (E′), loss modulus (E″), tan delta (δ), T_g_, and viscoelastic modulus. E′ represents the material’s ability to store elastic energy, while E″ measures its ability to dissipate energy as heat. Tan delta (δ) reflects the ratio of these components and characterizes the damping behavior of the material. DMA also detects changes in mechanical properties mainly related to the cross-linking density of the material, which affects its mechanical performance, including stiffness and elasticity. While T_g_ marks the transition from a rigid to a more flexible state, it is the cross-linking density that directly affects mechanical strength [18]. Higher cross-linking density generally correlates with increased T_g_ due to limited polymer chain mobility [18]. However, the same increasing trend in cross-linking density was observed in our study, as shown in Table 1.

**Figure 1:**
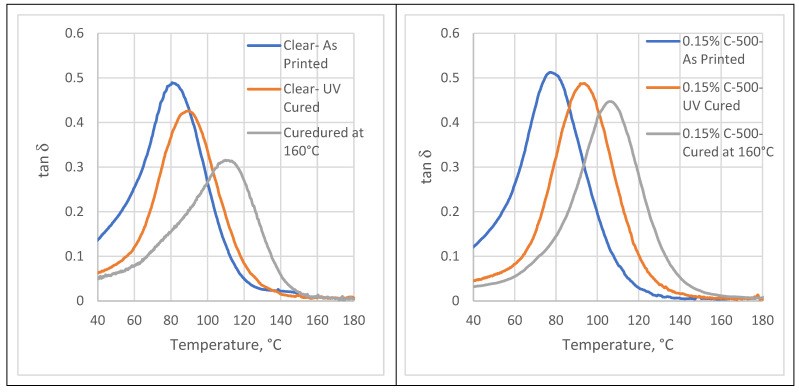
Tan delta curves for clear and nanocomposite samples with different post-curing methods.

Figure 1 displays tan δ curves for both clear and nanocomposite samples subjected to various post-curing methods. The storage modulus indicates the stiffness of the material, and the loss modulus is more indicative of its damping and energy dissipation capabilities, while the peak temperature indicates the T_g_. It is obvious that the stiffness values follow the following order: as printed < UV cured < cured at 160 °C (Table 1). This hierarchy is created by increased cross-linking density during curing. Increased cross-linking results in more covalent bonds between polymer chains, immobilizing segments and increasing the energy barrier to chain movement, resulting in tighter behavior and therefore higher stiffness and T_g_ values. Post-curing at 160 °C results in the highest T_g_ and stiffness values, reflecting the highest cross-link density in this post-curing process. The degree of cross-link density was also measured using Flory’s rubber elasticity theory equation (Equation (1)), and the data are recorded in Table 1.

(1) $$Crosslink\;Density=\frac{E^{\prime }}{3RT}$$

where E′ represents the storage modulus at the temperature T_g_ + 50 °C, R is the universal gas constant (8.314472 m^3^·Pa·K^−1^·mol^−1^), and T is the absolute temperature at T_g_ + 50 °C. Setting the temperature to T_g_ + 50 °C is widely used in polymer studies and applications to ensure that the material remains in its rubbery state well beyond the glass transition phase. This higher temperature helps to more clearly observe changes in the material’s properties, such as its elasticity, viscosity, and molecular mobility. At T_g_ + 50 °C, polymers are typically more flexible and easier to manipulate, which is crucial for certain processing techniques and experimental measurements [19].

The cross-linking density is consistent with the data from the tan δ curves and confirms that the cross-linking intensity increased in the following order: as printed < UV cured < cured at 160 °C. For nanocomposites compared to clear samples, the increments in cross-linking density are 14%, 7%, and 5% in the printed state, UV post-cured, and 160 °C post-cured, respectively. It can be concluded that the main factors influencing photopolymerization are the curing method and then the xGNP nanofiller. The mechanism of graphene nanofiller-induced polymerization in photopolymers involves the efficient absorption of photons by graphene nanofillers, leading to the generation of free radicals from photoinitiators and the subsequent initiation of the polymerization process [20]. The presence of xGNP nanofillers improves energy and charge carrier transfer, promotes more efficient polymerization kinetics, and facilitates the formation of a densely interconnected polymer network. The xGNP nanofillers also serve as nucleation sites for cross-linking between polymer chains, resulting in increased cross-linking density and improved mechanical properties of the nanocomposite material [21]. Overall, the incorporation of graphene nanofillers into photopolymer matrices improves the polymerization process and provides mechanical reinforcement, leading to the development of high-performance nanocomposite materials.

**Figure 2:**
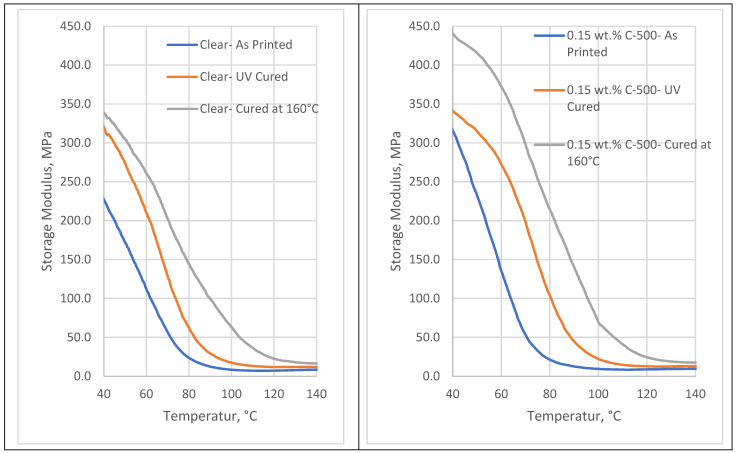
Storage modulus diagrams for clear and nanocomposite samples with different post-curing methods.

Figure 2 shows the superior storage modulus for xGNP-filled 3D-printed photopolymer nanocomposite samples compared to clear samples. The addition of xGNP nanofiller leads to an improvement in the storage modulus for printed and post-cured samples. It represents the ability of the material to store elastic energy when subjected to cyclic loading or deformation. The trend in the results is consistent with the elastic modulus of mechanical properties. The elastic modulus measures a material’s resistance to deformation under static loading conditions, while the storage modulus characterizes its stiffness under dynamic loading. It can be observed that post-curing at 160 °C leads to the highest storage modulus. This is due to the higher degree of cross-linking of these samples, as evidenced by the increased T_g_ values. The addition of xGNP also has a significant impact on the viscoelastic behavior of the nanocomposites, as evidenced by the storage modulus improvements observed with DMA. The xGNP nanoparticles act as reinforcements within the polymer matrix, improving the material’s ability to store and dissipate energy under dynamic loading conditions. This effect is visible even before post-curing, with a notable 39% improvement in bearing modulus for the printed samples. When combined with post-cure at 160 °C, these improvements are further optimized, suggesting that although cross-link density is increased by post-cure, the initial improvements are due to the presence of xGNP. This shows that xGNP plays a critical role in modifying viscoelastic properties from the outset, with post-curing serving to further refine these properties.

**Figure 3:**
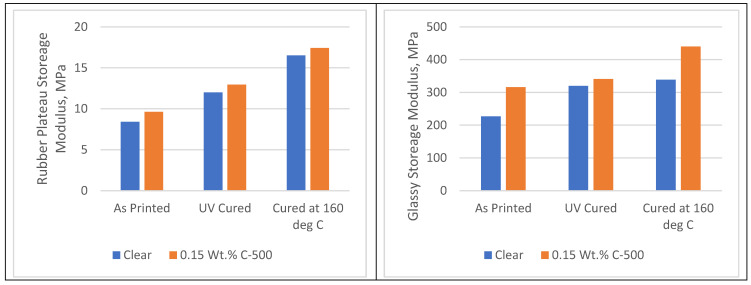
Rubber plateau and glassy storage moduli for clear and nanocomposite samples with different post-curing methods.

Figure 3 shows the improvement in the storage modulus of the rubber plateau modulus and the glassy modulus as a result of the addition of xGNP. The improvement in glassy storage modulus is more evident with 39% for as-printed samples, 6% for UV post-cured samples, and about 30% for 160 °C post-cured samples.

### 3.3. Thermal Properties

**Figure 4:**
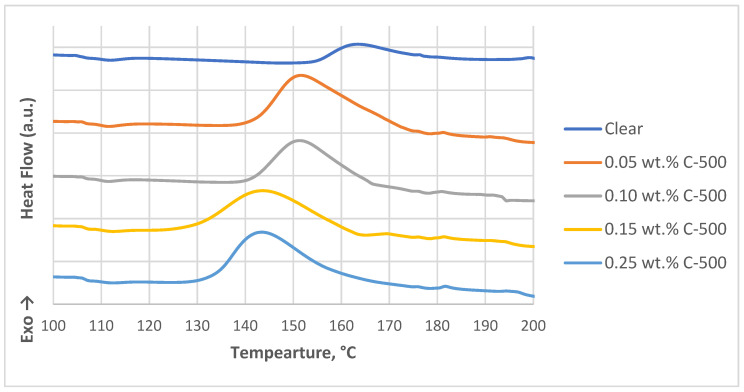
DSC thermograms for clear and C-500-filled photopolymer.

In this study, the DSC technique was used to monitor the curing degree of nanocomposites and clear resin. As shown in Figure 4, the DSC thermograms illustrate that for thermoset/nanofiller systems, the decrease in peak temperature in dynamic scans and the reduction in time at heat flow peak in isothermal scans were taken as evidence of acceleration in the early stage of the curing process. The area under the polymerization curve, which is larger for nanofiller mixtures compared with clear samples, indicates retardation of the crosslinking reaction due to limited UV penetration through the nanocomposite layers due to the presence of xGNP. These results are consistent with studies reporting similar effects in nanofiller systems, where increased filler-matrix interactions may slow rather than accelerate the curing process [22,23]. This is evident from the larger area under the polymerization curve for nanocomposite samples compared to clear samples, with a smaller area indicating a higher degree of cross-linking, suggesting a more complete cure. The reduced curing efficiency in nanocomposites is attributed to several mechanisms caused by graphene. Firstly, graphene acts as a physical barrier, disrupting the movement and interaction of reactive monomers, photoinitiators, or cross-linkers within the polymer matrix. Secondly, its exceptional thermal conductivity can result in uneven temperature distributions, potentially resulting in under-cured areas. Additionally, graphene’s UV shielding properties can prevent sufficient light penetration, further hindering curing in shielded areas and resulting in less efficient cross-linking. Moreover, the addition of nanofillers like xGNP shifts the exothermic peak from 163 °C to 143 °C in 0.25 wt% xGNP nanocomposite samples, which confirms the nucleation effect of xGNP and supports the curing process. The DSC thermogram data from Figure 4 summarized in Table 2 provides important insights into the curing behavior of the various samples containing different concentrations of graphene nanoplatelets (xGNP) C-500. The initial temperature (T_i_), peak temperature (T_p_), final temperature (T_f_), and heat of curing (ΔH) provide a comprehensive overview of how the xGNP concentration affects the thermal curing process of the nanocomposites.

The peak temperature represents the point of maximum reaction rate during the curing process. An increase in T_p_ from 145 °C in the clear sample to 165 °C in the 0.25 wt% C-500 sample indicates that higher xGNP concentrations require more energy to achieve the maximum curing reaction. This trend suggests that the presence of graphene nanoplatelets can increase the thermal stability of the system, which is likely due to enhanced interactions between the nanoplatelets and the polymer matrix. The heat of curing, which measures the total energy released during the cure reaction, shows a progressive increase with higher xGNP concentrations, ranging from 220 J/_g_ in the clear sample to 270 J/_g_ in the 0.25 wt% C-500 sample. This increase in ΔH suggests that higher xGNP concentrations contribute to a more complete or efficient curing reaction, possibly due to the catalytic effect of the nanoplatelets or improved thermal conductivity facilitating better heat distribution.

The initial and final temperatures of the peak provide additional information about the start and completion of the curing reaction. The shift of T_i_ and T_f_ to higher temperatures with increasing xGNP concentration could reflect the additional energy barrier created by the nanoplatelets, requiring more heat to initiate and complete the reaction. This broadening of the curing range suggests that xGNP not only affects the peak reaction conditions but also alters the overall reaction kinetics, potentially leading to a more controlled curing process.

**Figure 5:**
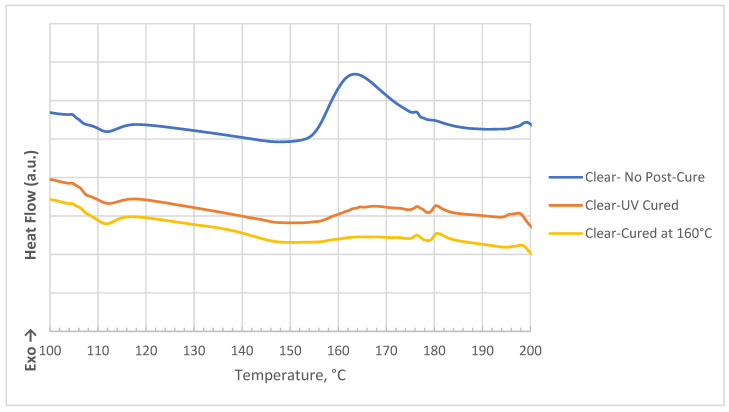
DSC thermograms for clear photopolymer with different post-curing processes.

The thermograms shown in Figure 5 demonstrate that complete curing of clear photopolymer samples can be achieved by post-curing processes that include either UV curing at room temperature or thermal curing at 160 °C. Each method uses different curing kinetics: UV curing initiates the polymerization process quickly, while thermal curing at 160 °C ensures that the polymer matrix takes longer to complete the reaction initiation, propagation, and termination phases. This extended time (post-curing) facilitates a more thorough and complete curing of the polymer, improving the final product’s cross-linking and stability.

**Figure 6:**
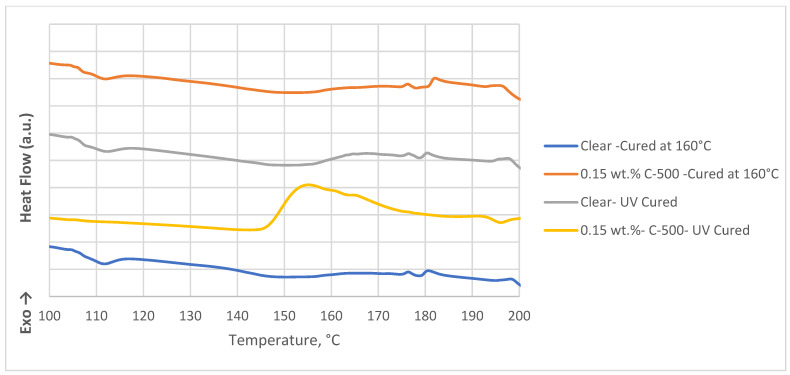
DSC thermograms for photopolymer nanocomposites with different post-curing processes.

Figure 6 presents DSC thermograms illustrating that UV post-curing does not fully complete the cross-linking reaction in xGNP photopolymer nanocomposites, whereas high-temperature curing at 160 °C achieves complete photopolymerization. UV curing is initiated when photoinitiators in the photopolymer absorb UV light, resulting in the formation of free radicals or cations that start the polymerization process. However, the presence of xGNP hinders the penetration of UV light, preventing a complete reaction and resulting in inadequate cross-linking with UV post-cure alone. In contrast, thermal curing does not rely on light and typically involves thermal initiators that decompose at high temperatures and produce free radicals. Alternatively, the heat can directly break some chemical bonds in the monomers, triggering the reaction. The elevated temperature also increases the mobility of monomers and polymer chains, increasing the likelihood of collisions that facilitate bond formation. This elevated kinetic energy allows reactions that are less likely at lower temperatures, creating a more cross-linked network. These results highlight the importance of considering nanofiller effects when optimizing the properties of SLA 3D-printed materials. Lopez et al., 2022 reported that graphene inhibits both thermally and UV-triggered polymerization [24].

### 3.4. Mechanical Properties of 3D-Printed Photopolymer Nanocomposites

**Figure 7:**
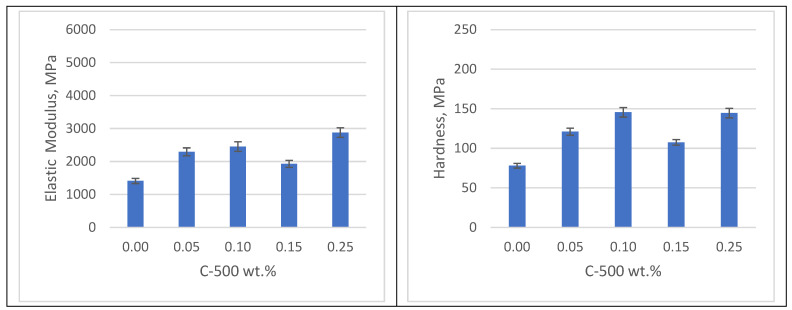
Mechanical properties of samples without post-curing.

**Figure 8:**
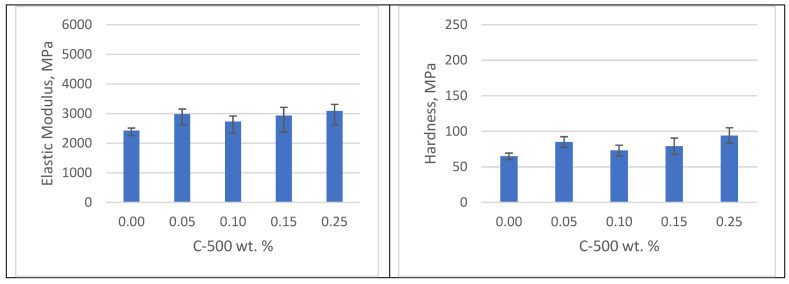
Mechanical properties of samples with UV post-curing.

**Figure 9:**
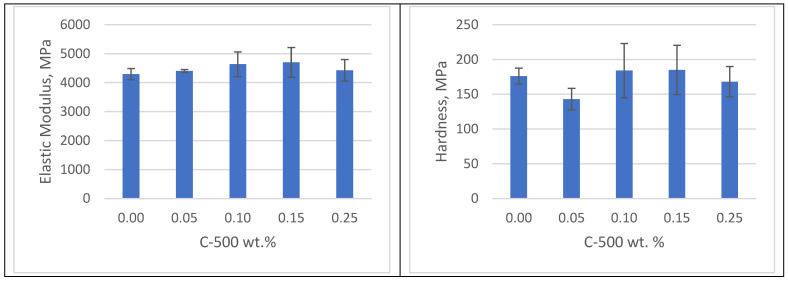
Mechanical properties of samples with curing at 160 °C.

Figure 7, Figure 8 and Figure 9 depict the elastic modulus and hardness of C-500 xGNP photopolymer nanocomposites with varying nanofiller ratios and post-curing conditions. The mechanical characterization of the 3D-printed nanocomposites was carried out using nanoindentation. As shown in Figure 7, the elastic modulus and hardness of the as-printed nanocomposites exhibit significant enhancement compared to clear photopolymer samples. Specifically, the 3D-printed nanocomposite with 0.25 wt% xGNP shows a remarkable improvement in elastic modulus by 104% and hardness by 85% compared to clear photopolymer samples. Hang et al. reported in 2019 that an effective method to increase photocatalytic efficiency and mechanical properties is the addition of nanoparticles (1–100 nm) to produce UV-curable coatings [25]. Recent studies have identified graphitic carbon nitride (g-C_3_N_4_) as a promising material for UV curing. g-C_3_N_4_ has similarities to graphene in that it is composed of carbon and nitrogen with some hydrogen impurities. It has a layered structure and numerous S-triazine functional groups on its surface. This configuration resembles a π-conjugated plane, similar to graphite, suggesting the possibility of inducing charge transfer and synergistic curing effects with initiators under UV irradiation [26].

C-500 xGNP photopolymer nanocomposites can only be printed with concentrations up to 0.25 wt% xGNP. This limited concentration is due to light absorption, which prevents sufficient light from penetrating the photopolymer layer to trigger the cross-linking reaction, thus preventing the layer-by-layer construction of nanocomposite samples.

Post-curing improves the mechanical properties of the photopolymer by promoting further cross-linking. As shown in Figure 8, after UV post-curing, the elastic modulus increases by approximately 27% and the hardness increases by 44%. UV post-curing increases the conversion of double bonds in the photopolymer, resulting in improved mechanical properties [27]. In addition, high-temperature post-curing at 160 °C enhances mechanical properties by facilitating cross-linking of uncured and partially cross-linked resin. Specifically, samples cured at 160 °C show only a slight improvement, with a 9% increase in elastic modulus and a 5% increase in hardness compared to clear samples treated with the same method, as shown in Figure 9.

The possible reason for the lower mechanical properties of xGNP photopolymer nanocomposites with UV curing compared to post-curing at 160 °C could be related to differences in the curing mechanisms and cross-linking density achieved by each method. UV curing requires photoinitiators to initiate the polymerization process, which may not fully penetrate or activate throughout the material, resulting in incomplete cross-linking and lower mechanical strength. In contrast, thermal curing typically requires higher temperatures, allowing for more extensive and uniform cross-linking throughout the material, resulting in enhanced mechanical properties. In addition, the UV curing process may result in more polymer chain scissions or other defects due to the high-energy radiation, which further contributes to lower mechanical performance compared to thermal curing [24].

## 4. Conclusions

(1) This study conclusively demonstrates that the integration of graphene nanoplatelets (xGNP) into SLA 3D-printed photopolymer nanocomposites significantly improves the mechanical and viscoelastic properties. The addition of xGNP ensures a significant improvement in the elastic modulus, hardness, and storage modulus through its reinforcing effect, which independently contributes to the performance of the printing material. Post-curing, particularly at high temperatures, maximizes these properties even further by increasing the cross-linking density. While post-curing is critical to achieving the highest mechanical performance, the fundamental role of xGNP addition is equally important.
(2) The incorporation of up to 0.25 wt% graphene nanoplatelets resulted in exceptional improvements, with elastic modulus and hardness increasing by 104% and 85%, respectively, compared to control samples. These improvements are primarily due to the uniform distribution and strong interaction of graphene within the photopolymer matrix, which also significantly improves viscoelastic properties, especially the storage modulus.
(3) Furthermore, the study reveals that by optimizing post-curing conditions such as UV curing and thermal curing at 160 °C, further tuning of these properties is possible by controlling the cross-linking density. The results highlight the innovative potential of graphene nanoplatelets to improve the performance of 3D-printed materials while highlighting the importance of post-processing techniques for achieving optimal material properties. This dual approach of xGNP integration and optimized post-cure enables the production of high-performance 3D-printed components, positioning this work at the forefront of advanced materials development for industries where improved mechanical properties are essential.

## 5. Future Scope

Future research on 3D-printed nanocomposites reinforced with graphene nanoplatelets could explore optimizing nanoplatelet concentrations beyond current limits to further improve material properties while maintaining printability. Investigating alternative nanofillers such as carbon nanotubes, nanoclays, or hybrid materials could provide comparative insights into mechanical, thermal, and electrical improvements. Evaluating the long-term performance and durability of these nanocomposites under various environmental conditions, such as temperature cycling, UV exposure, and mechanical stress, would help assess their suitability for real-world applications. Advanced characterization techniques, including in situ mechanical testing and real-time monitoring of the curing process, could provide deeper insights into the interactions between nanoplates and polymers. Expanding the manufacturing process for industrial applications in sectors such as automotive, aerospace, and biomedical devices, as well as research into sustainability and recyclability, could pave the way for environmentally friendly high-performance materials.

## Figures and Tables

**Table 1:** Viscoelastic properties of 3D-printed samples with different post-curing methods.

Formulations	Rubbery Modulus (MPa)	Glassy Modulus (MPa)	Cross-Link Density (mol/m^3^)
Clear-As Printed	8.42 ± 0.30	226.94 ± 8.00	816.9 ± 20
Clear-UV Cured	12.00 ± 0.40	320.12 ± 10.00	1164.7 ± 25
Clear-Cured at 160 °C	16.51 ± 0.50	338.67 ± 12.00	1602.3 ± 30
0.15 wt%C-500-As Printed	9.63 ± 0.35	315.87 ± 9.00	934.6 ± 22
0.15 wt%C-500-UV Cured	12.95 ± 0.45	341.01 ± 11.00	1256.5 ± 26
0.15 wt%C-500-Cured at 160 °C	17.43 ± 0.55	440.06 ± 14.00	1691.5 ± 35

**Table 2:** DSC Thermogram Summary for Figure 4.

Formulations	Initial Temperature (T_i_) [°C]	Peak Temperature (T_p_) [°C]	Final Temperature (T_f_) [°C]	Heat of Curing (ΔH) [J/_g_]
Clear	110	145	160	220
0.05 wt% C-500	115	150	170	240
0.10 wt% C-500	118	155	175	250
0.15 wt% C-500	120	160	180	260
0.25 wt% C-500	125	165	185	270

## Data Availability

Currently, the raw data critical to reproducing these results are not available for public sharing, as they are an essential part of an ongoing doctoral project. The raw data will be confidential until the project is completed and published.
